# Association of the MTHFR 677C>T and 1298A>C polymorphisms and male infertility risk: a meta-analysis

**DOI:** 10.1186/s12958-020-00649-1

**Published:** 2020-09-10

**Authors:** Fereshteh Aliakbari, Farkhondeh Pouresmaeili, Nahal Eshghifar, Zahra Zolghadr, Faezeh Azizi

**Affiliations:** 1 Men’s Health & Reproductive Health Research Center, Shahid Beheshti University of Medical Sciences, Tehran, Iran; 2 Department of Medical Genetics, Faculty of Medicine, Shahid Beheshti University of Medical Sciences, Tehran, Iran; 3 Department of Molecular and Cellular Biology, Faculty of Advanced Science and Technology, Tehran Medical Sciences, Islamic Azad University, Tehran, Iran; 4 Department of Biostatistics, school of allied medical Sciences, Shahid Beheshti University of Medical Sciences, Tehran, Iran; 5 Genetics Office, Non-Communicable Disease Control Department, Public Health Department, Ministry of Health and Medical Education, Tehran, Iran

**Keywords:** MTHFR, Polymorphisms, Male infertility

## Abstract

**Background and objectives:**

One of the possible male sterility risk factors are polymorphisms of Methylenetetrahydrofolate reductase (*MTHFR*). However, the epidemiologic investigations described inconsistent results regarding *MTHFR* polymorphism and the risk of male infertility. For that reason, we carried out a meta-analysis of published case-control studies to re-examine the controversy.

**Methods:**

Electronic searches of Cochrane, EMBASE, Google Scholar, and PubMed were conducted to select eligible studies for this meta-analysis (updated to May 2019). According to our exclusion and inclusion criteria, only high-quality studies that remarked the association between *MTHFR* polymorphisms and male infertility risk were included. The Crude odds ratio (OR) with a confidence interval of 95% (CI) was used to assess the relationship between MTHFR polymorphism and male infertility risk.

**Results:**

Thirty-four case-control studies with 9662 cases and 9154 controls concerning 677C/T polymorphism and 22 case-control studies with 5893 cases and 6303 controls concerning 1298A/C polymorphism were recruited. Both *MTHFR* polymorphisms had significant associations with male infertility risk (CT + TT vs. CC: OR = 1.37, 95% CI: 1.21–1.55, *P* = 0.00, I^2^ = 41.9%); (CC vs. CA + AA: OR = 0.82, 95% CI: 0.52–1.30, *P* = 0.04, I^2^ = 50.1%). Further, when stratified by ethnicity, the significant association results were observed in Asians and Caucasians for 677C/T and just Asians for 1298A/C.

**Conclusions:**

Some of *MTHFR* polymorphisms like MTHFR 677C > T are associated with an elevated male infertility risk. To confirm our conclusion and to provide more accurate and complete gene-environment communication with male infertility risk, more analytical studies are needed.

## Introduction

Infertility is a global problem and according to the World Health Organization, almost one in seven couples are affected by fertility complications [1, 2]. Male infertility is a heterozygous disorder caused by numerous genetic and environmental factors that lead to defects in spermatogenesis [3, 4]. This kind of fertility disorder accounts for 20–50% of causes. According to studies, there is a positive correlation between serum folate concentrations, density, and normal morphology of sperm [5]. Therefore, the Folate pathway is likely to be important in male fertility [6]. Methylenetetrahydrofolate reductase (MTHFR) is a key enzyme in folate metabolism and plays a vital role in balancing the storage of methyl groups between DNA synthesis and its methylation [7]. DNA methylation is one of the important epigenetics features that play an essential role in regulating gene expression in spermatogenesis [8]. The mutations of C677T and A1298C in the MTHFR gene reduce the enzyme activity and cause male sterility in some populations [9].

The catalytic enzyme- encoding gene MTHFR is located at the end of the short arm of chromosome 1 (1p36.3) and has 33 exons [10].

Two C677T and A1298C polymorphisms that significantly alter MTHFR enzyme activity are recognized in this gene [11]. The conversion of cytosine into thymine as a result of a point mutation in nucleotide 733 in exon 4 of the MTHFR gene results in the replacement of alanine by valine [12]. This point mutation leads to the formation of an unstable and heat-sensitive MTHFR enzyme with low activity. Due to the decreased enzymatic activity of mutant MTHFR, increased serum homocysteine levels are achieved. The MTHFR enzyme has a 19% function in homozygotes and 71% in heterozygotes compared to normal people [13].

The adenine to cytosine conversion at nucleotide position 3289 in exon 3 of the MTHFR gene (A1298C), also leads to the replacement of glutamine by alanine [14]. There are few studies on A1298C polymorphism; however, it has been shown that CC genotype has an equivalent function to 79% of the AA genotype. Homozygotes do not show high serum homocysteine levels for the A1298C allele. But individuals with combined heterozygote A1298C and C677T polymorphisms have biochemical characteristics similar to C677T homozygotes with elevated levels of homocysteine and decreased levels of folate [15, 16]. Numerous studies have examined the association between MTHFR polymorphism and male infertility, but the conclusions are argumentative [17]. The reason for this can be attributed in part to the ethnicity differences. There are only four meta-analyses that have evaluated the effect of MTHFR C677T polymorphism on male infertility in Asians [18].

The N. Gupta and colleagues studied the Indian population [18], Wiener’s worked on the men’s idiopathic infertility in Russian population [19], Z. Ren and colleagues studied the Chinese population [20], and V. Rai and P. Kumar research [21] focused on the relationship between one type of MTHFR A1298C and male infertility.

In this study, with the help of eligible findings, we carried out a meta-analysis to provide a comprehensive assessment of the association between C677T and A1298C MTHFR polymorphisms with male infertility.

## Materials and methods

### Literature search

A comprehensive literature quest in Cochrane, EMBASE, Web of Science, PubMed, Scopus, as well as Google Scholar databases was conducted for all articles regarding the impact of C677T and A1298C polymorphisms on male infertility published up to May 2019“ Include from when the analysis was done. Articles were obtained with the following keywords: “methylenetetrahydrofolate reductase” or “MTHFR”, ‘polymorphism” or “variant”, “677C > T”, “1298A > C” and “male infertility”. The inclusion criteria were: 1) Well defined case-control study design; 2) sufficient data for examining an odds ratio (OR) with 95% confidence interval (CI). The exclusion criteria were: 1) non-human studies; 2) articles not available in English and Farsi languages; 3) duplicate study by the same group with lower sample number; 4) cases only studies; v) insufficient genotyping data.

### Data extraction

According to the inclusion and exclusion criteria, data extraction was achieved by two independent investigators. Any disagreements of the studies were resolved through a comprehensive reassessment by the other author and only high- quality studies can be included in our meta-analysis. Reference lists of all included full-text manuscripts were screened for additional articles. Authors of papers were contacted to ask clarification where inadequate information was provided. The following data were collected from studies: first author, year of publication, ethnicity, sample size, allele distribution in cases and controls and, a genotyping method used. The different ethnic groups were classified as Caucasian and Asian.

### Statistical analysis

All analyses were performed using STATA 14.1 software (Stata Corporation, College Station, TX, USA). In this research, *P*- values were calculated two-sided and *P* = 0.05 was statistically considered as significant. The Hardy–Weinberg equilibrium (HWE) was calculated by the Chi-square test in control groups, to verify the representation of the study population. The correlation between the polymorphisms and male infertility risk was calculated via assessment of odds ratios (ORS) and 95% confidence interval (CI). Pooled ORs and their 95% CIs for dominant, codominant, and recessive inheritance models were calculated.

The significance of the pooled OR was assessed by Z-test and *P* < 0.05 (Forest plot). To indicate the presence of heterogeneity, the random effect model was selected; otherwise, the fixed-effects model was chosen.

Of all the models available, a funnel plot was designed to assess the publication bias, and an asymmetrical plot was considered a sign of these impresses. The degree of the asymmetry in these plots was measured with the help of Egger’s test and a *p*-value less than 0.05 was introduced as a significant publication bias. Sensitivity analysis was conducted to measure the effect by ignoring a single study at a time.

## Results

### Study characteristics

According to the present investigation, for both polymorphisms, 137 relevant articles were identified.

**Fig. 1:**
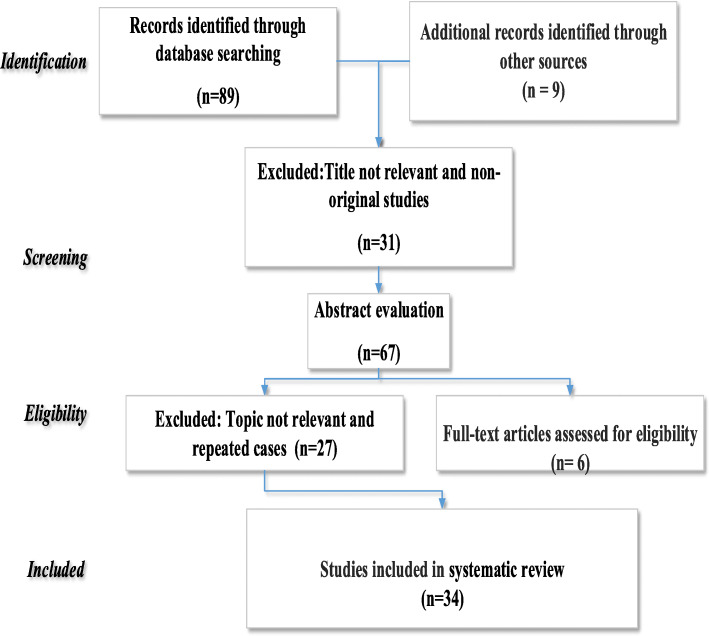
PRISMA flow chart for selecting relevant articles of MTHFR 677C/T

The process of literature retrieval and selection is shown in Fig. 1. There were 34 case-control studies with 9662 cases and 9154 controls concerning 677C/T polymorphism and 22 case-control studies with 5893 cases and 6303 controls concerning 1298A/C polymorphism. Also, for the 677C/T polymorphism, there were 23 studies of the Asian population and ten studies of the Caucasian population, and for1298A/C polymorphism, fifteen and five, respectively. The specifications and data for the considered studies are summarized in Table 1.

**Table 1:** Summary of the included studies

NO.	Author (Year)	Country	Ethnicity	Case/Control	Genotyping method	HWE^a^
1	Bezold 2001 [22]	Germany	Caucasian	255/200	NA	Yes
2	L. Stuppia 2003 [23]	Italy	Caucasian	93/105	PCR-RFLP	Yes
3	Ebisch 2003 [24]	Netherlands	Caucasian	77/113	PCR-RFLP	Yes
4	Park 2005 [25]	Korea	Asian	373/396	PCR-RFLP	Yes
5	Lee 2006 [26]	China	Asian	373/396	PCR-RFLP	Yes
6	Raman 2005 [27]	India	Asian	151/200	PCR-RFLP	Yes
7	Paracchini 2006 [28]	Italy	Caucasian	59/46	PCR-RFLP	Yes
8	Taioli 2006 [28]	Italy	Caucasian	59/56	PCR-RFLP	Yes
9	Lee 2006 [26]	Korea	Asian	360/325	PCR-RFLP	Yes
10	ZC 2007 [29]	China	Asian	355/252	PCR-RFLP; Sequencing	Yes
11	Zhang 2007 [29]	China	Asian	355/252	PCR-RFLP	Yes
12	Dhillon 2007 [30]	India	Asian	179/200	PCR-RFLP	Yes
13	Tetik A 2008 [31]	Turkey	Asian	100/50	Real-Time PCR	Yes
14	Ravel 2009 [32]	France	Caucasian	252/114	PCR-RFLP	Yes
15	Farcas 2009 [33]	Romania	Caucasian	66/67	PCR-RFLP	Yes
16	Yang 2010 [34]	Australia	Caucasian	131/29	NA	Yes
17	Rejender 2011 [18, 35]	India	Asian	522/315	Sequencing	Yes
18	Safarinejad 2011 [36]	Iran	Asian	164/328	PCR-RFLP	Yes
19	Gava 2011 [37]	Brazil	Latin	156/233	Real-Time PCR	Yes
21	Vani 2012 [38]	India	Asian	266/230	PCR-RFLP	Yes
22	Eloualid 2012 [39]	Spain	Caucasian	344/690	PCR-RFLP	Yes/No
23	Balkan 2013 [40]	Turkey	Asian	108/125	Real-Time PCR	Yes
24	Stangler 2013 [41]	Slovenia	Caucasian	100/111	Multiplex PCR	Yes
25	Sadiq 2014 [42]	Jordan	Asian	150/150	PCR-RFLP	Yes
26	Mahdi 2014 [43]	India	Asian	637/364	PCR-RFLP	Yes
27	Colagar 2014 [10]	Iran	Asian	118/132	PCR-RFLP	Yes
28	Jiang 2014 [44]	China	Asian	215/133	NA	Yes
29	Weiner 2014 [45]	Russia	Caucasian	271/301	Multiplex PCR	Yes
30	Kurzawski 2015 [6]	Poland	Caucasian	284/352	Real-Time PCR	Yes
31	Jiang 2015 [46]	China	Asian	296/204	SNaPshot multiplex system	Yes
32	Momenzadeh 2015 [9]	Iran	Asian	131/130	PCR-RFLP	Yes
33	Irfan 2016 [22]	Pakistan	Asian	437/218	PCR-RFLP	Yes
34	Wang 2017 [47]	China	Asian	1759/1826	PCR-RFLP	Yes
35	Najafipour 2017 [48, 49]	Iran	Asian	280/120	Sequencing	Yes
36	Mazhar 2018 [50]	Pakistan	Asian	232/114	PCR-RFLP	Yes/No
37	Murphy 2011 [51]	Swede	Caucasian	153/184	competitive allele-specific PCR	Yes

^a^Hardy–Weinberg equilibrium

### MTHFR 677C > T

**Fig. 2:**
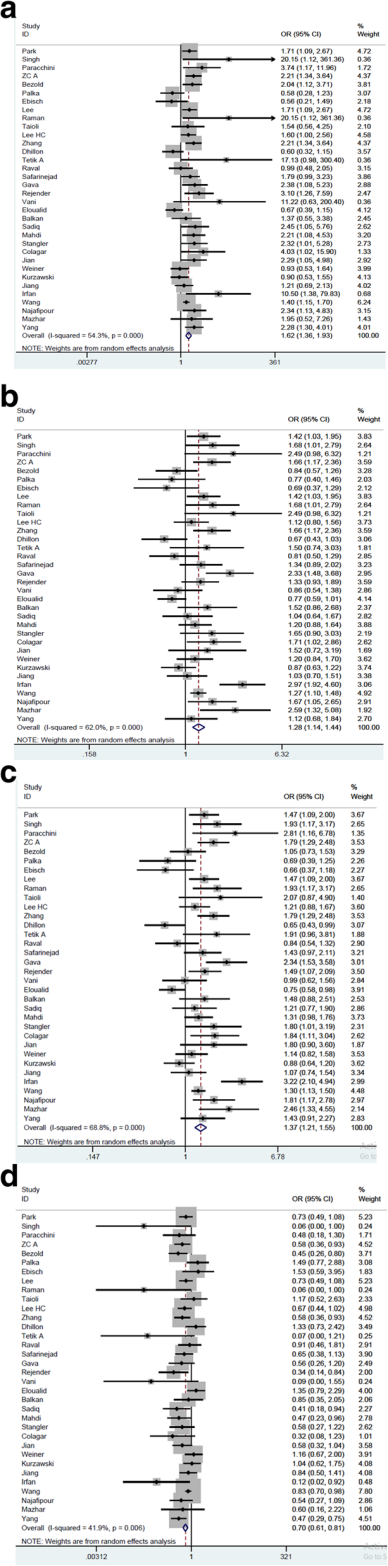
Forest plot of male infertility risk associated with MTHFR 677C > T polymorphism [**a** for TT vs. CC; **b** for TC vs. CC; **c** for TT + TC vs. CC; **d** for TT vs. TC + CC]. The *squares and horizontal lines* correspond to the study-specific OR and 95% CI. The *area of the squares* reflects the weight (inverse of the variance). The *diamond* represents the summary OR and 95% CI

Overall, we realized that the 677C > T polymorphism was associated with the risk of male infertility. In this study, the frequency of the TT genotype to CC showed a significant increase (*P* = 0.00). Also, the frequency of TC genotype to CC, the frequency of TT genotype to the combination of TC + CC genotypes, and the frequency of TT + TC combined genotype to CC showed a significant increase (*P* = 0.006) (Table 2, Fig. 2).

**Table 2:** Main results for the MTHFR 677C > T polymorphism in the meta-analysis

Variables	Cases/Controls	TT vs. CC		TC vs. CC		TT + TC vs. CC		TT vs. TC + CC	
		OR(95% CI)	*P* ^*^	OR (95% CI)	*P* ^*^	OR (95% CI)	*P* ^*^	OR (95% CI)	*P* ^*^
**Total**	**9662/9154**	**1.62 (1.36, 1.93)**	**0.00**	**1.28 (1.14, 1.44)**	**0.00**	**1.37 (1.21, 1.55)**	**0.00**	**0.70 (0.61, 0.81)**	**0.00**
**Ethnicity**	**Cases/Controls**	**TT vs. CC**		**TC vs. CC**		**TT + TC vs. CC**		**TT vs. TC + CC**	
		**OR(95% CI)**	***P*** ^*^	**OR (95% CI)**	***P*** ^*^	**OR (95% CI)**	***P*** ^*^	**OR (95% CI)**	***P*** ^*^
**Asian**	**7989/6887**	**1.78 (1.48–2.16)**	**0.01**	**1.35 (1.20–1.53)**	**0.00**	**1.46 (1.29–1.65)**	**0.00**	**0.66 (0.56–0.77)**	**0.08**
**Caucasian**	**1673/2267**	**2.23 (0.84–1.80)**	**0.00**	**1.02 (0.83–1.27)**	**0.03**	**1.08 (0.86–1.36)**	**0.00**	**0.87 (0.63–1.20)**	**0.00**

^*^P value of the chi- square test for heterogeneity

When the results were stratified by ethnicity, the following positive associations were also observed in the Caucasian population where TT genotype had significantly elevated frequency to the CC genotype (*P* = 0.002), TC had significant increased frequency to CC (*P* = 0.038), TT frequency to the TC + CC genotypes (*P* = 0.007), and TT + TC showed higher frequency than CC genotype (*P* = 0.005). In addition, we found that 677C/T polymorphism was significantly associated with the male infertility risk in the Asian population where the TT to CC genotype (*P* = 0.01), TC to CC (*P* = 0.002), TT to TC + CC (*P* = 0.084), and combination of TT + TC genotype frequencies to the CC (*P* = 0.00) showed significant difference.

### MTHFR 1298A/C

**Fig. 3:**
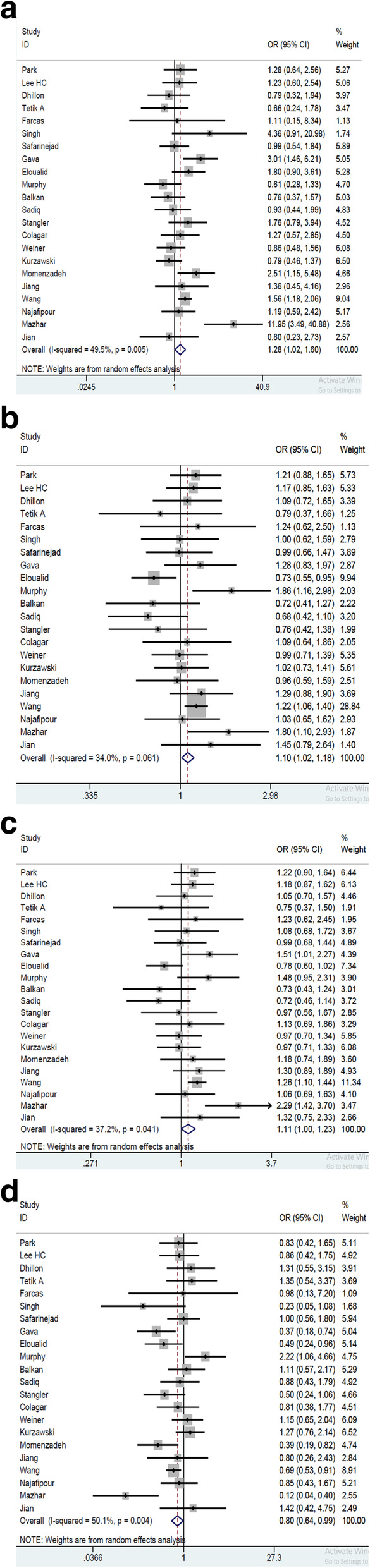
Forest plot of male infertility risk associated with MTHFR 1298A/C polymorphism [**a** for CC vs. AA; **b** for CA vs. AA; **c** for CC + CA vs. AA; **d** for CC vs. CA + AA]. The *squares and horizontal lines* correspond to the study-specific OR and 95% CI. The *area of the squares* reflects the weight (inverse of the variance). The *diamond* represents the summary OR and 95% CI

We attained that the 1298A/C polymorphism was not associated with risk of male infertility where the frequency of CC to the AA genotype (*P* = 0.09), CA to the AA (*P* = 0.210), CC to the CA + AA (*P* = 0.194), and CC + CA to the AA (*P* = 0.084) genotype was not significantly different, Table 3, Fig. 3. When the analysis was stratified by ethnicity, the subsequent negative associations were also observed in Caucasian population: CC to AA genotype (*P* = 0.530), CA to AA genotype (*P* = 0.167), CC to CA + AA genotypes (*P* = 0.405), and CC + CA genotype to AA (*P* = 0.237) did not show significant increase in the frequency. Also, in Asian population, there was no significant difference between the examined genotypes as the frequency of CC to AA (*P* = 0.168); CA to AA (*P* = 0.071); CC to CA + AA (*P* = 0.305), and CC + CA to AA (*P* = 0.073) was observed in the population.

**Table 3:** Main results for the MTHFR 1298A/C polymorphism in the meta-analysis

Variables	Cases/Controls	CC vs. AA		CA vs. AA		CC + CA vs. AA		CC vs. CA + AA	
		OR(95% CI)	*P* ^*^	OR (95% CI)	*P* ^*^	OR (95% CI)	*P* ^*^	OR (95% CI)	*P* ^*^
**Total**	**5893/6303**	**1.23 (0.97, 1.55)**	**0.002**	**1.08 (0.96, 1.20)**	***p*** **= 0.031**	**1.10 (0.99, 1.24)**	**0.014**	**0.86 (0.68, 1.08)**	**0.000**
**Ethnicity**	**Cases/Controls**	**TT vs. CC**		**TC vs. CC**		**TT + TC vs. CC**		**TT vs. TC + CC**	
		**OR(95% CI)**	***P*** ^*^	**OR (95% CI)**	***P*** ^*^	**OR (95% CI)**	***P*** ^*^	**OR (95% CI)**	***P*** ^*^
**Asian**	**4918/4782**	**1.23 (0.92, 1.64)**	**0.010**	**1.11 (0.99, 1.25)**	**0.227**	**1.13 (0.99, 1.30)**	**0.050**	**0.87 (0.67, 1.13)**	**0.006**
**Caucasian**	**1065/1521**	**1.21 (0.96, 1.53)**	**0.258**	**0.88 (0.74, 1.05)**	**0.370**	**0.91 (0.78, 1.07)**	**0.671**	**0.82 (0.52, 1.30)**	**0.096**

^*^P value of the chi- square test for heterogeneity

### Evaluation of heterogeneity

**Fig. 4:**
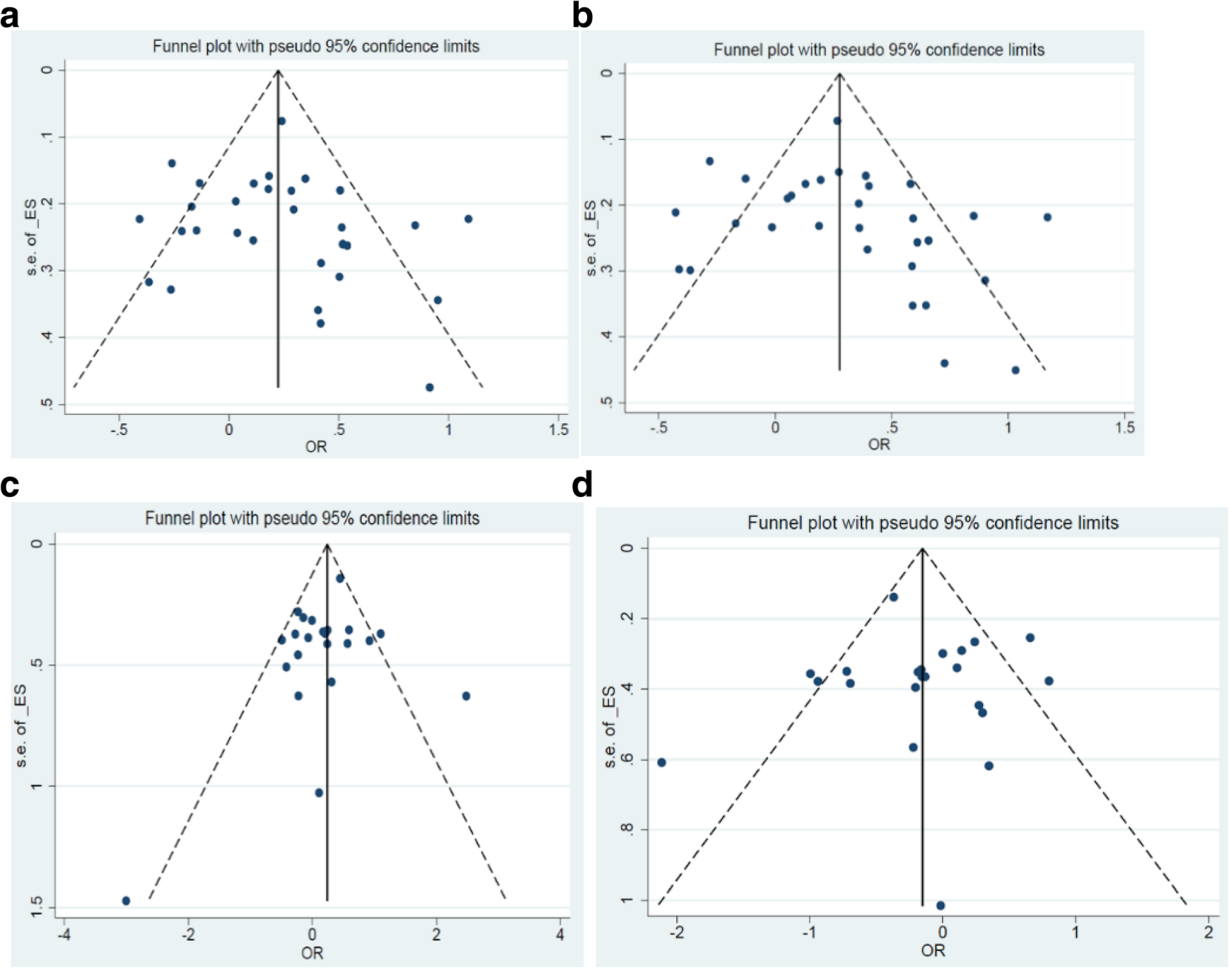
Funnel plot for publication bias test [for 677C > T polymorphism: **a** for TC vs. CC, *p = 0.281*; **b** for TT + TC vs. CC, *p = 0.196*; for 1298A > C polymorphism: **c** for CC vs. AA, *p = 0.403*; **d** for CC vs. CA + AA, *p = 0.235*]. Each point represents a separate study for the indicated association. Log [or], natural logarithm of OR. Horizontal line, mean effect size

We used Egger’s test and Begg’s funnel plot to assess the publication bias. The shape of funnel plots has shown in Fig. 4, and in most cases funnel plot symmetry was observed. Also, the statistical results confirmed the results of the plots. For the 677C/T polymorphism, the heterogeneity was reckoned between each of the studies using the chi-square test, *p*-value of 0.024 for TT vs. CC; *p*-value = 0.281 for TC vs. CC; *p*-value = 0.196 for the dominant model; and *p*-value = 0.008 for the recessive model, respectively. For the 1298A/C polymorphism, the heterogeneity was reckoned between each of the studies using the Chi-square test, *p*-value of 0.403 for CC vs. AA; *p*-value = 0.235 for CA vs. AA; *p*-value = 0.200 for dominant model; and *p*-value =0.235 for recessive model, respectively.

### Sensitivity analysis

In the sensitivity analysis, the effect of each survey on the pooled OR was assayed by repeating the meta-analysis while deleting each study, one at a time. This method verified the stability of our total results.

### Publication bias

Begg’s funnel plot and Egger’s test were conducted to assess the publication bias of the literature. The shape of funnel plots reveals a bit of evidence of funnel plot asymmetry (Fig. 4). The statistical results still show publication bias (for 677C > T polymorphism: *p*-value = 0.02 and *p*-value =0.00 for 1298A/C).

## Discussion

Male infertility is a disorder that is affected by environmental and genetic factors. MTHFR gene plays a crucial role in folate metabolism as a result of spermatogenesis. Studies in different populations have shown contradictory results for the association between MTHFR gene polymorphisms and male infertility [52, 53]. The present meta-analysis from 34 published studies, including 9662 cases and 9154 controls for 677C/T and 22 published case-control studies with 5893 cases and 6303 controls for1298A/C, explored the association between two potentially functional polymorphisms in the MTHFR gene and male infertility risk. The numbers of included studies in this meta-analysis are much higher than the prior meta-analyses which dramatically increased the statistical power of the analysis due to the low number of pieces of evidence. Overall, we found that the variant genotypes of the MTHFR 677C/T were significantly associated with male infertility risk. In stratified analysis by ethnicity, we found that the variant genotype of the MTHFR 677C/T polymorphism was significantly associated with the risk of male infertility in the Caucasian and Asian populations.

On the other hand, there was no significant association between the variant genotype of the MTHFR 1298A/C polymorphism and the risk of male infertility in Caucasian and Asian populations.

In a meta-analysis study by H. Nikzad, et al. 2015, an MTHFR 677C/T polymorphism showed a significant association between allelic, dominant and codominant models and the risk of male infertility (*P* < 0.001) [54]. Concerning 677C/T variation, our results were consistent with other meta-analysis results (M. Gong, et al. [55], F. Tüttelmann, et al. [56], N. Gupta, et al. [18], and W. Wu, et al. [57], but in contradiction with the results of B. Wei, et al. [58] and A.S.Weiner, et al. [19].

In another study, B. Wei, et al. [58] showed that both 677C/T and 1298A/C polymorphisms were not significantly associated with male infertility risk. However, in the classified analysis by ethnicity, it was reported that 677C / T polymorphism had a significant association with the risk of male infertility in the Asian population. However, the results of our studies were in contrast to the results of B. Wei, et al. meta-analysis and showed a clear relationship between both polymorphisms and male infertility. The reasons for these contradictions between the mentioned studies could be the presence of other unknown causal genes and/or their variations in combination to some environmental factors which may strongly influence male infertility, ethnic differences, selection bias, and different matching criteria.

In recent years, new genetics techniques such as GWAS have been used to evaluate the genetic variation of many diseases, including infertility, especially in idiopathic cases [55]. Concerning MTHFR common polymorphism and its association with male infertility, two studies have been conducted by Aston et al. and the results are different from our study [59, 60]. The reasons for this difference are:(1) The small sample size in the GWAS studies makes it difficult to interpret; (2) Our study includes the totality of studies conducted in various populations, while in Aston studies, only European population have been investigated.

Some of the limitations in this article were: lack of sufficient studies for the African and Latin American populations, unadjusted estimates, bias publication, and heterogeneities for MTHFR polymorphisms among all the studies. Although some modest bias could not be eliminated, this meta-analysis suggests that the MTHFR 677 T and 1298C alleles might be effective risk factors for male infertility, especially in the Asian population. We suggest screening genetic test for the MTHFR SNP’s as new male infertility biomarkers for Asian people.

Also, nutritional management and folate administration under proper medical care could lessen the risk of infertility in such populations.

In summary, this meta-analysis supports the hypothesis that both MTHFR polymorphisms, MTHFR 677C > T and MTHFR 1298A/C, might be markers of male infertility susceptibility, especially in the Asian population. However, more comprehensive studies are warranted to validate our findings.

## Conclusion

Our meta-analysis results showed that the MTHFR 677C > T polymorphism was associated with an enhanced risk of male infertility, and supporting the hypothesis that the most common MTHFR polymorphisms may be a potential cause of male infertility.

However, there was no relation between MTHFR 1298A/C and male sterility. Subsequent investigations should use standardized unbiased genotyping methods, homogeneous infertility patients, well-matched controls, and subgroup analysis according to the sperm concentration to confirm our findings in the future.

## Data Availability

The original data from the survey is available.

## Abbreviations

*MTHFR*: Methylenetetrahydrofolate reductase
CI: Confidence interval
OR: Odds ratio
HWE: Hardy–Weinberg equilibrium
